# Correlation between peri-implant bone mineral density and primary implant stability based on artificial intelligence classification

**DOI:** 10.1038/s41598-024-52930-7

**Published:** 2024-02-06

**Authors:** Yanjun Xiao, Lingfeng Lv, Zonghe Xu, Lin Zhou, Yanjun Lin, Yue Lin, Jianbin Guo, Jiang Chen, Yanjing Ou, Lin Lin, Dong Wu

**Affiliations:** 1 School and Hospital of Stomatology, Fujian Medical University, Fuzhou, 350001 China; 2 Fujian Provincial Engineering Research Center of Oral Biomaterial, Fujian Medical University, Fuzhou, 350001 China; 3 Newland Digital Technology Co., Ltd., Fuzhou, Fujian China; 4 Research Center of Dental and Craniofacial Implants, Fujian Medical University, Fuzhou, 350001 China

**Keywords:** Outcomes research, Biomedical engineering

## Abstract

Currently, the classification of bone mineral density (BMD) in many research studies remains rather broad, often neglecting localized changes in BMD. This study aims to explore the correlation between peri-implant BMD and primary implant stability using a new artificial intelligence (AI)-based BMD grading system. 49 patients who received dental implant treatment at the Affiliated Hospital of Stomatology of Fujian Medical University were included. Recorded the implant stability quotient (ISQ) after implantation and the insertion torque value (ITV). A new AI-based BMD grading system was used to obtain the distribution of BMD in implant site, and the bone mineral density coefficients (BMDC) of the coronal, middle, apical, and total of the 1 mm site outside the implant were calculated by model overlap and image overlap technology. Our objective was to investigate the relationship between primary implant stability and BMDC values obtained from the new AI-based BMD grading system. There was a significant positive correlation between BMDC and ISQ value in the coronal, middle, and total of the implant (*P* < 0.05). However, there was no significant correlation between BMDC and ISQ values in the apical (*P* > 0.05). Furthermore, BMDC was notably higher at implant sites with greater ITV (*P* < 0.05). BMDC calculated from the new AI-based BMD grading system could more accurately present the BMD distribution in the intended implant site, thereby providing a dependable benchmark for predicting primary implant stability.

## Introduction

The formation of strong osseointegration is very important for the success of dental implants. Excessive fretting of the implant during the formation of osseointegration will lead to the formation of fibrous tissue between the implant and the bone, thereby impeding the implant's healing process. When fretting exceeds 100 µm, it substantially heightens the risk of implant failure^1^. The osseointegration requires a certain primary implant stability^2^. Primary implant stability refers to the mechanical stability formed immediately after implantation. The primary implant stability is associated with multiple factors, including bone density and quality, macro- and micro- design of the dental implant, and the surgical procedure^3,4^.

Primary implant stability can be reflected by many indexes, such as implant insertion torque (IT), implant stability quotient (ISQ), periotest value (PTV), among others. Previous studies have established a correlation between the bone mineral density (BMD) of the implant site and insertion torque value (ITV) as well as ISQ^5^. Therefore, preoperative treatment planning should include an analysis of the BMD at the intended implant site to predict primary implant stability, forming the basis for a reasonable surgical procedure. The widely accepted bone quality classification proposed by Lekholm and Zarb^6^ employs preoperative radiographs to assess the amount of visible cortical and trabecular bone, resulting in four distinct scores^7^. Shahlaie et al.^8^ reported that this bone classification system was inaccurate, as it was possible to differentiate between bone types 1 and 4, whereas bone types 2 and 3 might be more difficult to classify. Misch et al.^9^ introduced a classification system based on tactile feedback and Hounsfield unit (HU) images of bone during implantation surgery. Their approach utilized tactile feedback to replicate the sensation of drilling bone during dental implantation. Additionally, Greenstein et al.^10^ compared bone to oak and other materials, and divided bone into 4 types according to the hole preparation feel of a 2 mm twist drill. Despite these classification systems, they tend to be subjective and rely on localized BMD assessment, often lacking coverage for the clinical diversity encountered in practice. Clinical practice often encounters variations in jawbone density at implant sites along the proximo-distal and buccolingual directions. Hence, it is necessary to refine the BMD distribution further and evaluate it accurately. In addition, there has been a notable lack of insufficient literature supporting the impact of BMD on ISQ and ITV in various implant sites, such as the coronal and apical.

**Figure 1:**
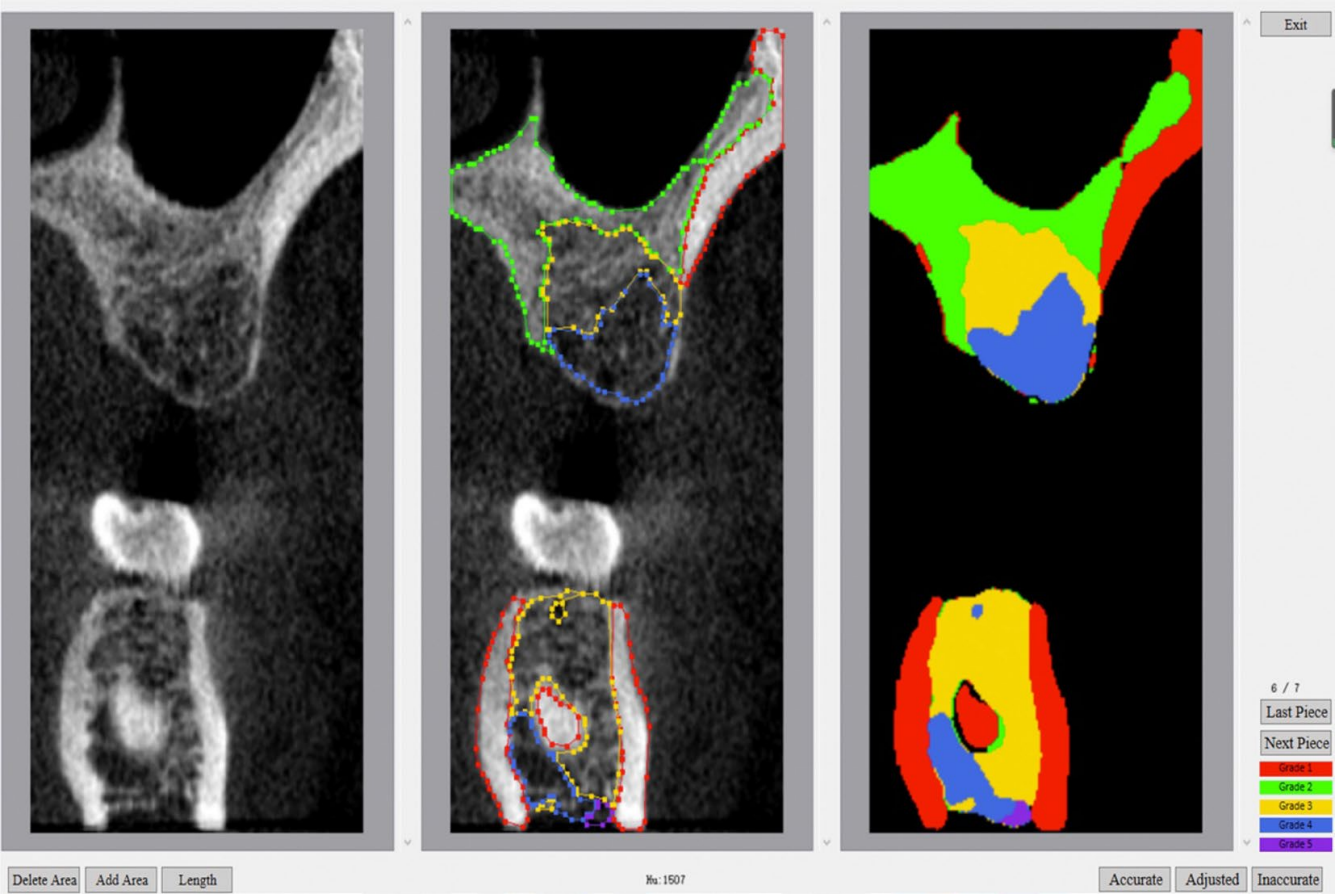
This is a Figure Schemes follow the same formatting.

In our previous studies, we preliminarily established a new artificial intelligence (AI) based BMD grading system^11^, which introduced a fresh approach to BMD classification. According to HU value, BMD was divided into five grades: 1 grade for HU value > 1000, 2 grade for HU value 700–1000, 3 grade for HU value 400–700, 4 grade for HU value 100–400, and 5 grade for HU value < 100. After the cone beam computed tomography (CBCT) section, the system can automatically grade the jaw BMD through artificial intelligence analysis and interpretation (Fig. 1). In this study, we integrated the new AI-based BMD grading system with implant design software to investigate the correlation between BMD at the coronal, middle or apical of the 1 mm alveolar bone site around the implant and the ISQ and ITV.

## Materials and methods

**Figure 2:**
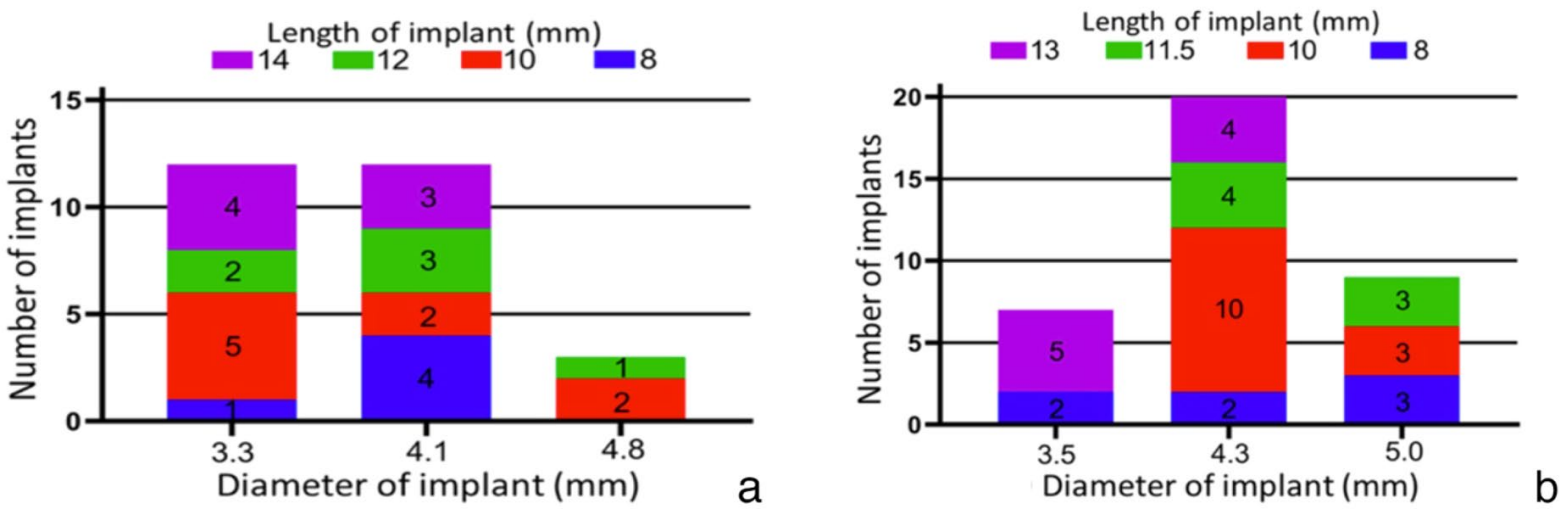
(**a**) Straumann Implant distribution. (**b**) Nobel implant distribution.

Patients diagnosed with dental defects eligible for dental implantation were recruited from the Department of Implantation during the period spanning August 2021 to January 2022. Following stringent inclusion and exclusion criteria, a total of 49 patients with 63 implants were enrolled, including 27 Straumann BL implants and 36 Nobel Replace PMC implants, as illustrated in Fig. 2.

Inclusion criteria: (a) Patients with dentition defects (over 18 years old); (b) Healthy periodontal tissue without cyst, tumor, other bone destruction, and history of orthodontic treatment; (c) Alveolar bone width and height sufficient for routine implantation; (d) Normal preoperative blood examination results, including normal bleeding and coagulation function and platelet counts; (e) Patients had good compliance.

Exclusion criteria: (a) Contraindications for implant surgery; (b) Hematological diseases such as leukemia, aplastic anemia, taking anticoagulants or blood coagulation tests in the past 12 weeks; (c) Inadequate alveolar bone width and height necessitating bone augmentation procedures or resulting in alveolar bone plate fractures or deletions; (d) Severe periodontitis; (e) Systemic diseases or medication use that may affect soft and hard tissue healing; (f) Uncontrolled systemic diseases such as hypertension and diabetes.

**Figure 3:**
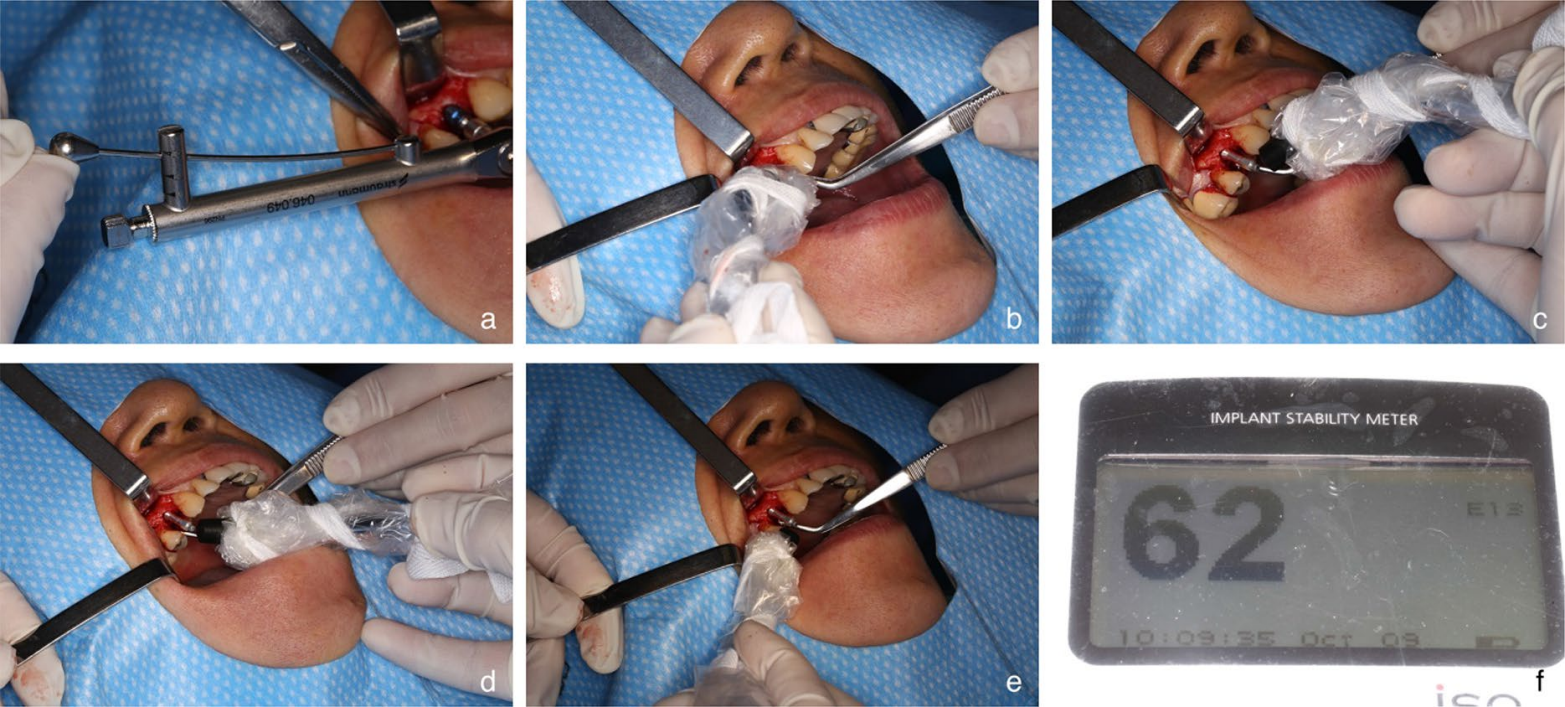
(**a**) Measurement of insert torque with a torque wrench. (**b**–**e**) Using a resonance frequency analyzer to measure the ISQ value of the implant from four directions. (**f**) The values were displayed on the ISQ Instrument.

Measurement and intraoperative data (Fig. 3). (a) Insert torque measurement: insert torque was recorded and categorized as either ≥ 35 N/cm or < 35 N/cm, employing a torque wrench provided within the corresponding surgical kit; (b) Resonant frequency analysis: Osstell ISQTM (Osstell, Integration Diagnostic AB, GoteborgSvagen, Sweden) was used to measure the implant stability in ISQ, by a skilled operator. (c) Note: It is imperative to acknowledge that SmartPeg should be utilized solely within a single treatment session for a given patient. The measurement probe was meticulously aligned perpendicular to the long axis of the SmartPeg. The lip (cheek), tongue (palate), mesial, and distal sites were measured 3 times each, and the results were presented as the average value for each site. (d) All measurements were done by the same dentist. The study design received approval from the Ethics Committee of Biomedical Research of the School and Hospital of Stomatology, Fujian Medical University (Date 8.9.2021/No.60). Informed consent was obtained from each patient after providing detailed explanations regarding the study's content and purpose.

**Figure 4:**
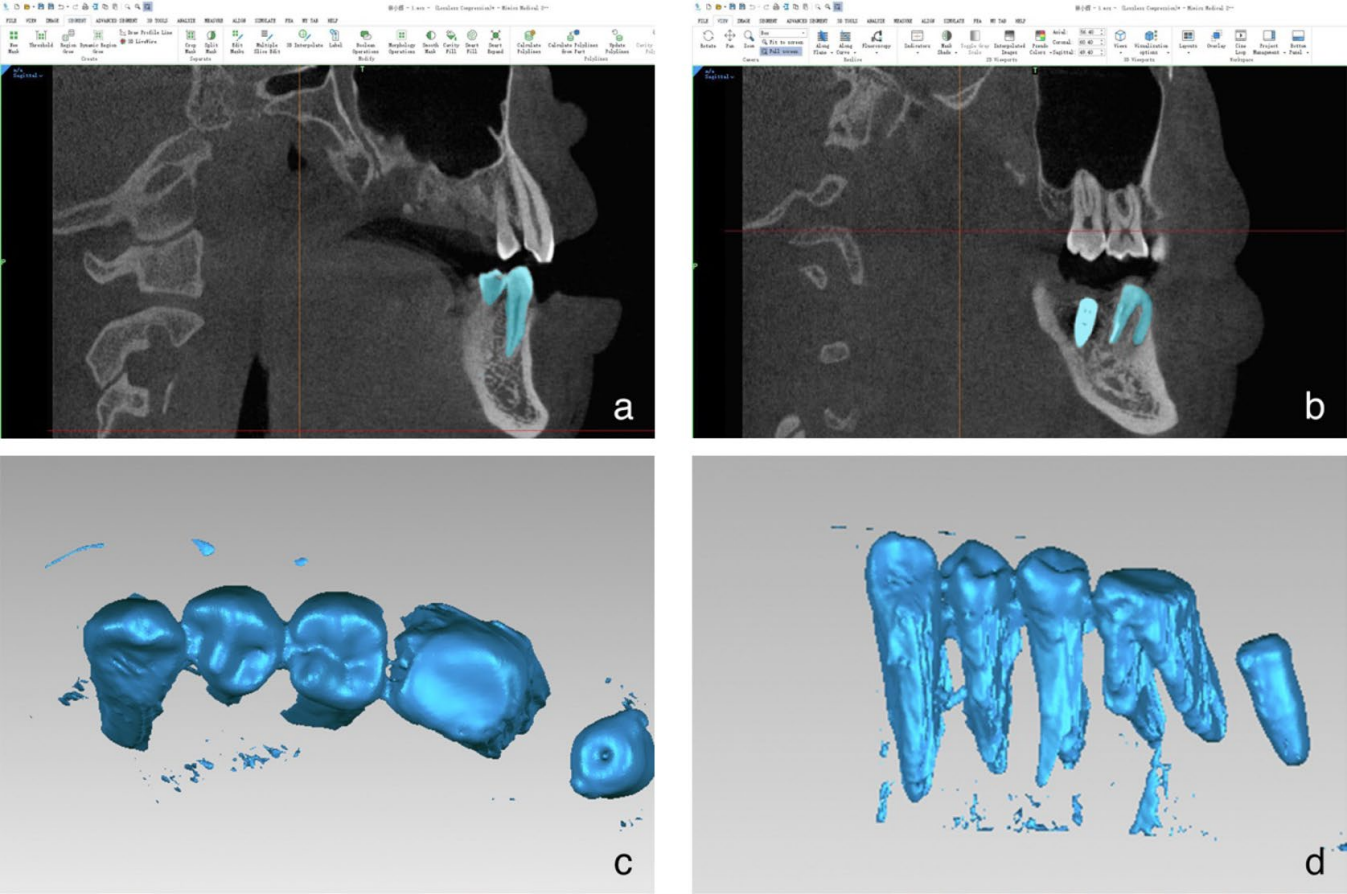
(**a**,**b**) Using Mimics 21.0 software for modeling. (**c**,**d**) Extracted 3D models of implants and adjacent natural teeth.

As implant artifacts may affect the identification of BMD in CBCT images, postoperative implant positions were restored to the preoperative CBCT, and AI-based BMD grading system results surrounding the implants in the preoperative CBCT were investigated. The process entailed the following steps:

The postoperative CBCT was converted in Digital Imaging and Communications in Medicine (DICOM) format and subsequently imported into Mimics 21.0 software. The threshold value was adjusted to separate the implant from 2 to 4 adjacent teeth and other tissues. The three- dimensional (3D) model of the implants and adjacent teeth were extracted (Fig. 4) and exported in STereoLithography (STL) format to obtain accurate actual implant positions on the preoperative CBCT.

**Figure 5:**
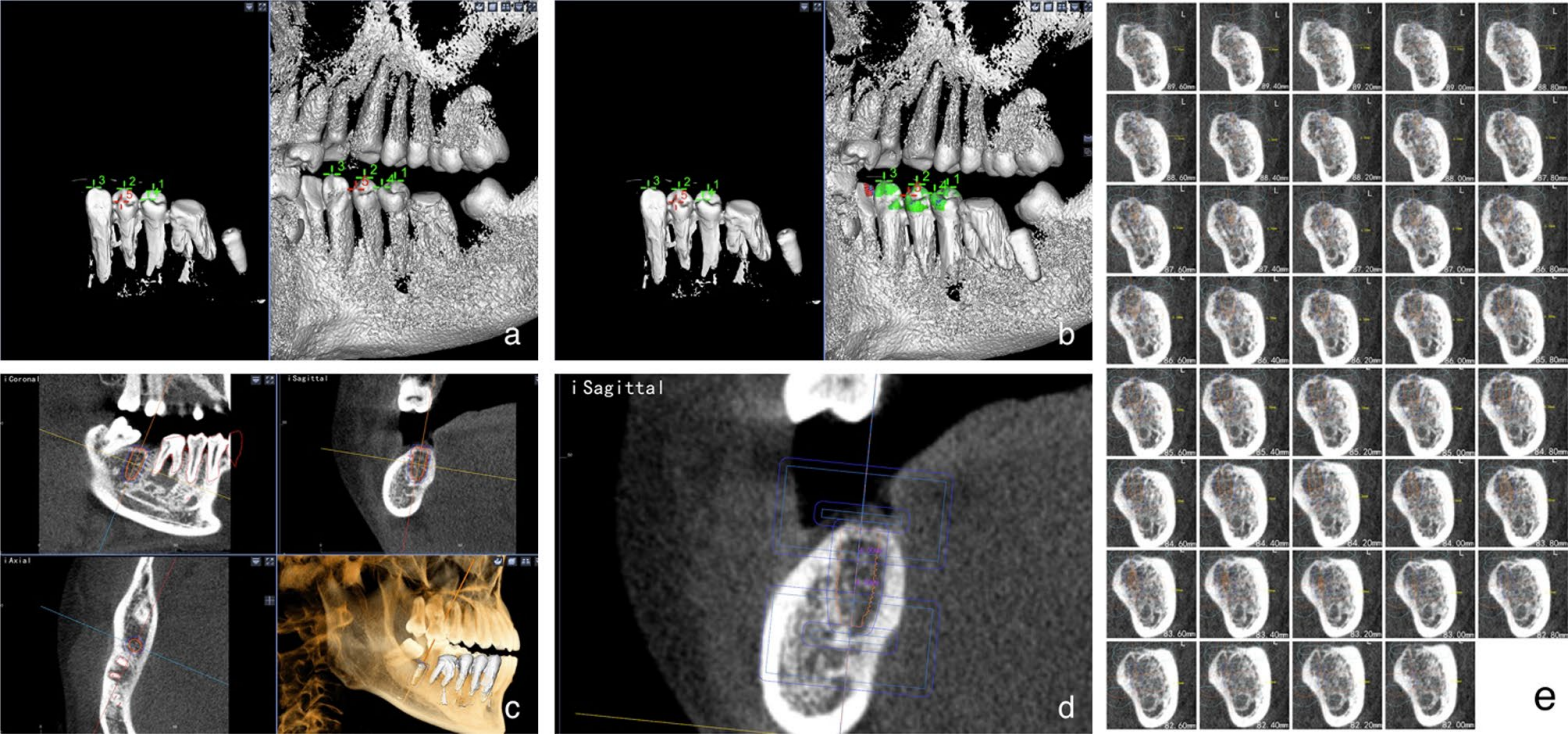
(**a**) Set registration points based on the cusps. (**b**) Registered and displayed the postoperative implant position. (**c**) Virtual implant placement based on actual implant. (**d**) Divided the implant into three equal parts. (**e**) Captured each CBCT layer image on the coronal plane.

Using the implant design software, we opened the preoperative CBCT data and imported the postoperative dentition along with the implanted 3D model. We then established 4–6 registration points based on the dental cusps (Fig. 5a), followed by a precise registration and matching of the postoperative 3D model data with the preoperative CBCT data. This allowed for the accurate display of the postoperative implant position on the preoperative CBCT (Fig. 5b). The virtual implant was placed according to the actual implant model and position (Fig. 5c). Inserted 4 markers, determined the position of the coronal, middle, and apical trisection of the implant (Fig. 5d), measured the length of about 8–10 mm in the blank site as the ruler, and captured a screenshot of each layer in the coronal plane with the minimum slice thickness (0.2 mm) of CBCT as the unit (Fig. 5e).

In the implant design software, opened the preoperative CBCT data, imported the postoperative dentition and implanted 3D model, set 4–6 registration points according to the cusps (Fig. 5a), registered and matched the postoperative 3D model data with the preoperative CBCT. This allowed for the accurate display of the postoperative implant position on the preoperative CBCT (Fig. 5b). Subsequently, the virtual implant was strategically positioned in accordance with the actual implant model and its location (Fig. 5c). 4 markers were inserted to ascertain the positions of the coronal, middle, and apical trisections of the implant (Fig. 5d). A measurement of approximately 8–10 mm was taken in the blank site to serve as a reference scale. Subsequently, screenshots were taken of each layer in the coronal plane, utilizing the minimum slice thickness (0.2 mm) of the CBCT as the standard unit of measurement (Fig. 5e).

**Figure 6:**
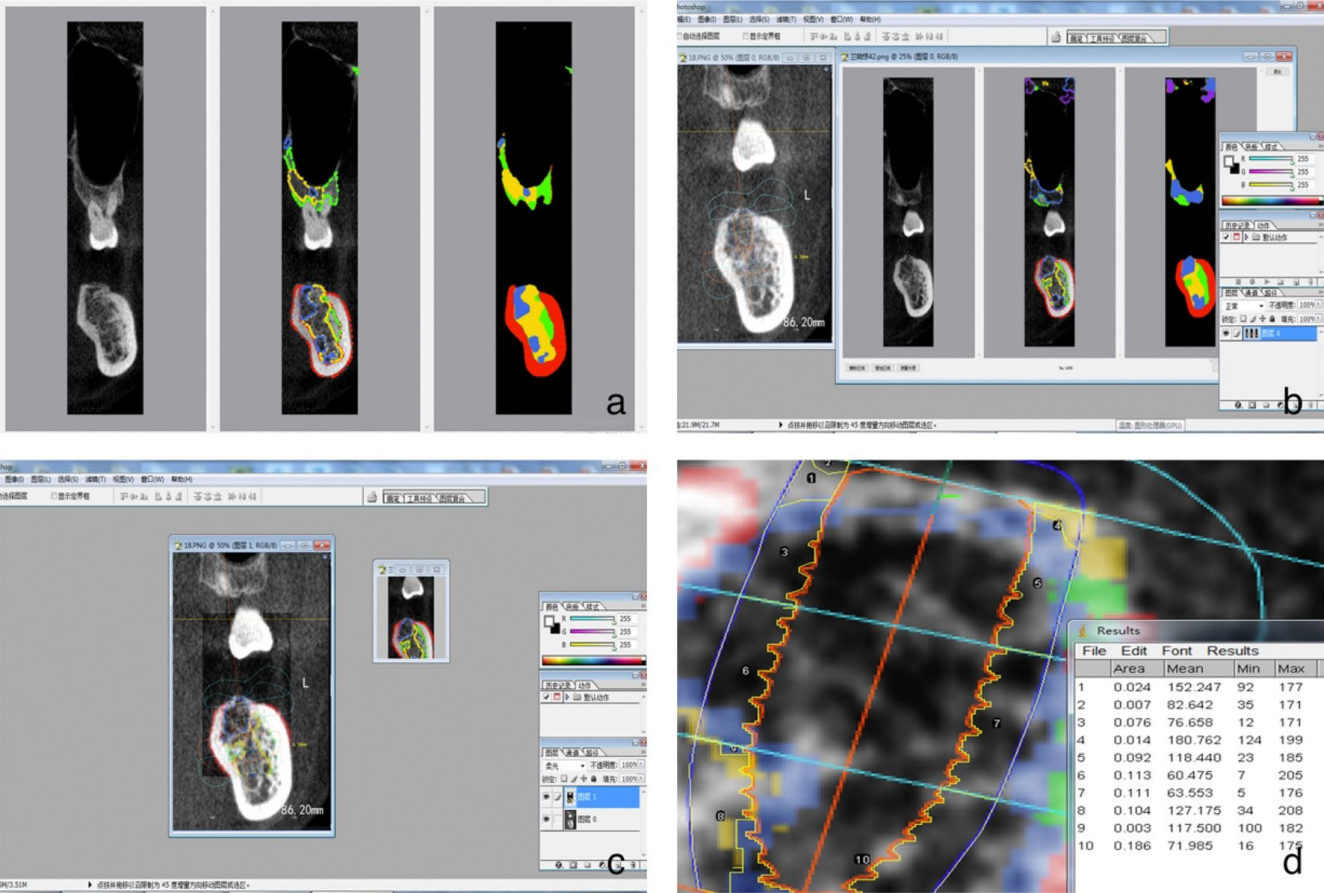
(**a**) Obtained the BMD distribution on each layer of DicomReader on a coronal plane. (**b**,**c**) Used Photoshop CS 6.0 software to overlap the image of the implant design of the same section with that of DicomReader. (**d**) Used ImageJ software to measure the proportion of BMD distribution of different parts around the implant on all sections.

The patient's preoperative CBCT data was imported into DicomReader software. By slicing through the edentulous site on the coronal plane, we were able to employ an AI-based Bone Mineral Density (BMD) grading system. This innovative system automatically identified the bone density distribution image. Screenshot tool was employed to capture a screenshot of each layer using the minimum CBCT slice thickness (0.2 mm) as the unit (Fig. 6a).

Using Photoshop CS 6.0, the implant design screenshots from the corresponding coronal sections were overlapped on those from the DicomReader software. Adjusting transparency to ensure the clear visualization of both the virtual implant and the surrounding bone density distribution (Fig. 6b). Overlapping all screenshots on the coronal section of the same implant site (Fig. 6c). To facilitate statistical analysis, bone mineral density coefficients (BMDC) were introduced to assign values to BMD. The method employed was as follows: Image J software was used to calculate the proportion of each type of bone in different parts of each layer within 1 mm around the virtual implant in CBCT. The proportions were classified as Grade 1 (P1) through Grade 5 (P5) (Fig. 6d), with an additional category for areas without alveolar bone labeled as blank (P0). Given that Grade 1 represented the highest BMD and Grade 5 the lowest, BMD was scored accordingly: 5 for Grade 1, 4 for Grade 2, 3 for Grade 3, 2 for Grade 4, and 1 for Grade 5. The BMDC was calculated based on the proportion of bone in each grade, represented as BMDC = 5 × P1 + 4 × P2 + 3 × P3 + 2 × P4 + 1 × P5 + 0 × P0. The BMDC of the coronal, middle, apical and total were calculated.

Spss22.0 statistical software was used to input all clinical data and establish a database. For the data that did not conform to the normal distribution, the correlation between two data was analyzed using the Spearman correlation test, the difference test of two independent samples used Mann–Whitney test, and the difference test of multiple independent samples used Kruskal–Wallis test. Data were expressed by median and quartile spacing.

### Ethical approval

This study was conducted in accordance with the ethical principles of the Declaration of Helsinki. Approval was granted by the Ethics Committee of Biomedical Research of the School and Hospital of Stomatology, Fujian Medical University (Date 8.9.2021/No.60).

### Informed consent

Informed consent was obtained from all individual participants included in the study.

## Results

### Correlation analysis between BMDC around implant and ISQ

**Figure 7:**
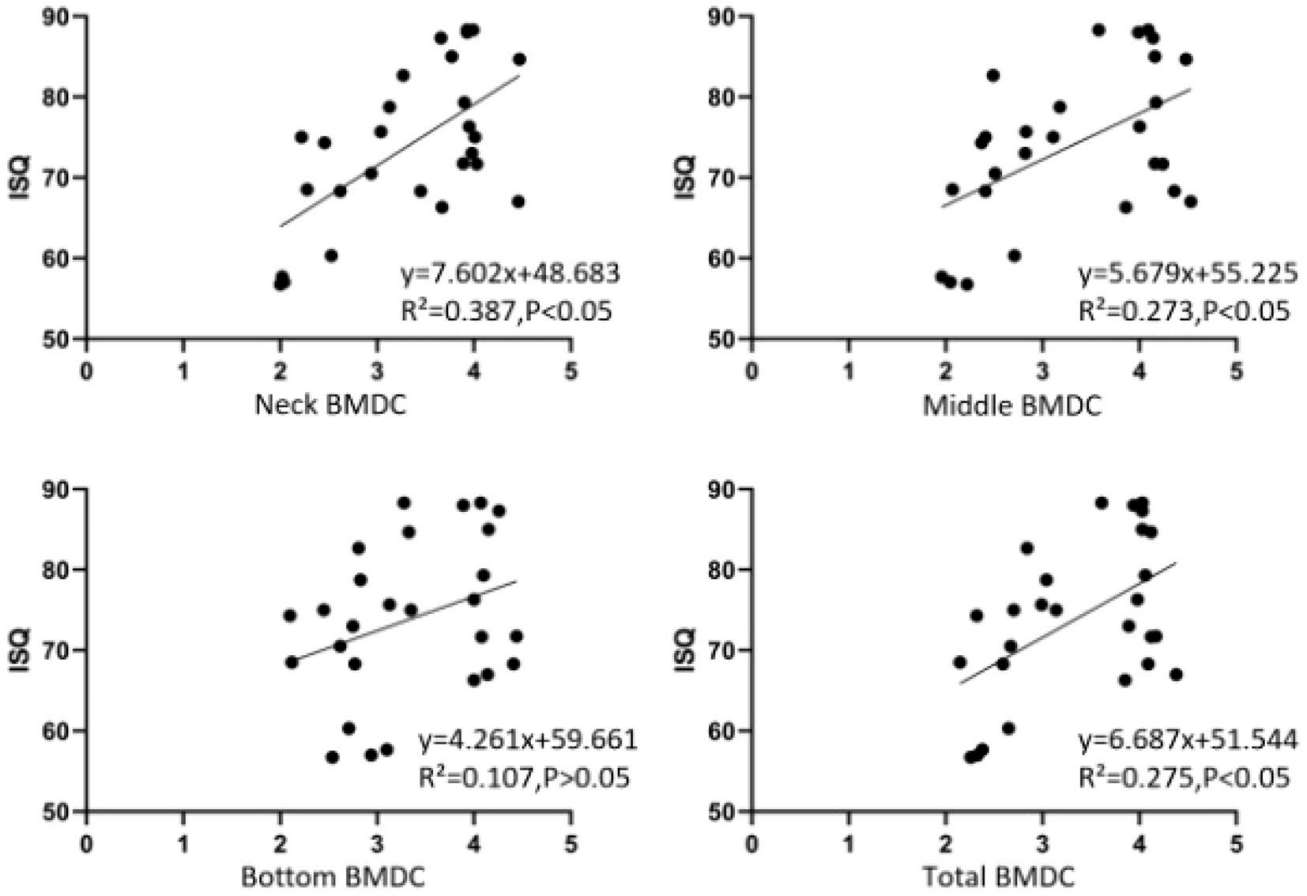
Scatter plots of ISQ and BMDC at different sites in Straumann implant group.

Scatter plots were constructed to assess the relationship between Implant Stability Quotient (ISQ) and Bone-to-Implant Contact Density (BMDC) at different sites (coronal, middle, apical, and total) in Straumann implant Group (Fig. 7) The regression equations were obtained as follows:

- y = 7.602x + 48.683 (x for Neck BMDC, y for ISQ, R2 = 0.387, *P* = 0.001, < 0.05).
- y = 5.679x + 55.225 (x for Middle BMDC, y for ISQ, R2 = 0.273, *P* = 0.005, < 0.05).
- y = 4.261x + 59.661 (x for Bottom BMDC, y for ISQ, R2 = 0.107, *P* = 0.097, > 0.05).
- y = 6.687x + 51.544 (x for Total BMDC, y for ISQ, R2 = 0.275, *P* = 0.005, < 0.05).

Due to the non-normal distribution of the data, Spearman correlation analysis was used to ascertain the relationship between ISQ and BMDC at each site of Straumann implants. The correlation between ISQ and BMDC at each site of the Straumann implant group in Table 1 showed that the BMDC in the coronal of the implant was significantly and positively correlated with ISQ; the BMDC in the middle of the implant was significantly and positively correlated with ISQ; the BMDC at the apical of the implant was not significantly correlated with ISQ; the total BMDC around the implant was significantly and positively correlated with ISQ.

**Table 1:** Correlation between ISQ and BMDC at each site of Straumann implant group.

Site	N	Spearman correlation analysis	
		R	*P*
Coronal BMDC	27	0.511	0.006
Middle BMDC	27	0.413	0.032
Apical BMDC	27	0.295	0.135
Total BMDC	27	0.415	0.031

**Figure 8:**
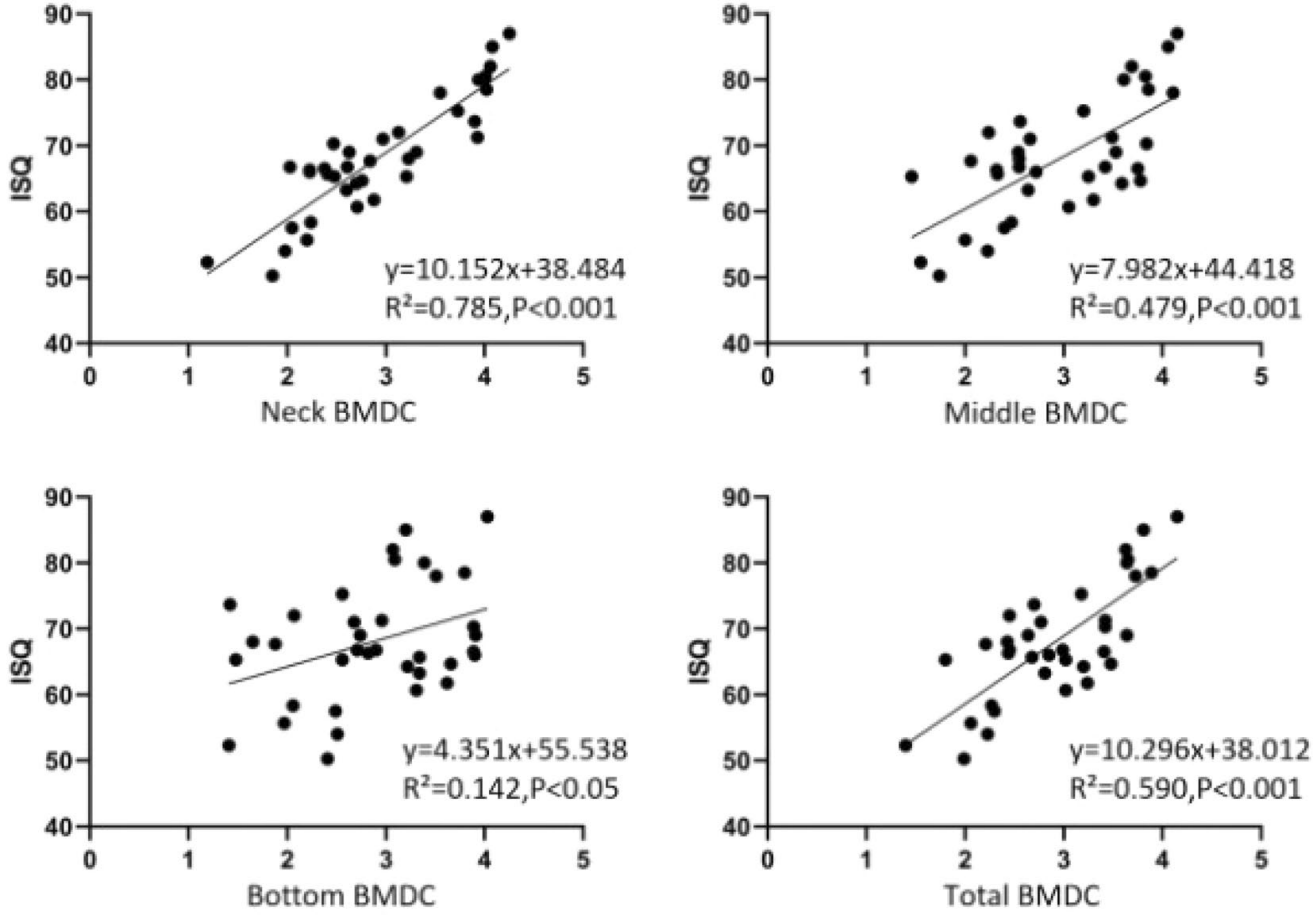
Scatter plots of ISQ and BMDC of different sites in Nobel implant group.

In the Nobel implant group, the scatter plots of ISQ and coronal, middle, apical and total BMDC after implantation in the Nobel implant group were presented in Fig. 8. The regression equations were obtained as follows:

- y = 10.152x + 38.484 (x for Neck BMDC, y for ISQ, R2 = 0.785, *P* < 0.001).
- y = 7.982x + 44.418(x for Middle BMDC, y for ISQ, R2 = 0.479, *P* < 0.001).
- y = 4.351x + 55.538 (x for Bottom BMDC, y for ISQ, R2 = 0.142, *P* = 0.023, < 0.05).
- y = 10.296x + 38.012 (x for Total BMDC, y for ISQ, R2 = 0.590, *P* < 0.001).

Since the data did not follow the normal distribution, Spearman correlation analysis was used to calculate the correlation between the BMDC of each site of Nobel implant and ISQ, respectively. The correlation between ISQ and BMDC of each site of Nobel implant group in Table 2 showed that the BMDC at the coronal of the implant was significantly and positively correlated with ISQ; the BMDC in the middle of the implant was significantly and positively correlated with ISQ; the BMDC at the apical of the implant was not significantly correlated with ISQ; and the total BMDC around the implant was significantly and positively correlated with ISQ.

**Table 2:** Correlation between ISQ and BMDC at each site of Straumann implant group.

Site	N	Spearman correlation analysis	
		R	*P*
Coronal BMDC	36	0.838	< 0.001
Middle BMDC	36	0.635	< 0.001
Apical BMDC	36	0.312	0.064
Total BMDC	36	0.689	< 0.001

### Difference in BMDC with different ITV

Given the non-normal distribution of our data, Spearman correlation analysis was used to determine the relationship between ITV and BMDC at different sites of both implant groups.

In the Straumann implant group, the difference in BMDC at different sites of the group under different ITV was shown in Table 3. The resluts indicated that the BMDC in coronal with ITV ≥ 35 N/cm was higher than that with ITV < 35 N/cm, and this difference was statistically significant. However, the BMDC was not significantly different in the middle, apical, and total regions around implants, regardless of ITV.

**Table 3:** Difference in BMDC at different Straumann implant group sites under different ITV.

Site	ITV < 35 N/cm	ITV ≥ 35 N/cm	Mann–Whitney U test	
	N = 9	N = 18	Z	*P*
Coronal BMDC	2.53 (2.03, 3.69)	3.83 (3.08, 3.98)	− 2.109	0.035
Middle BMDC	2.71 (2.14, 4.17)	3.72 (2.50, 4.16)	− 1.183	0.237
Apical BMDC	3.13 (2.82, 4.08)	3.30 (2.72, 4.11)	− 0.154	0.877
Total BMDC	2.70 (2.35, 4.06)	3.87 (2.79, 4.04)	− 1.029	0.304

In the Nobel implant group, the difference in BMDC at different sites of the group under different ITV in Table 4 indicated that the coronal BMDC, the middle BMDC and total BMDC in the ITV ≥ 35 N/cm group were higher than those in the ITV < 35 N/cm group, and the differences were statistically significant. There was no statistical difference in the apical BMDC between the two groups.

**Table 4:** Difference in BMDC at different Nobel implant group sites under different ITV.

Site	ITV < 35 N/cm	ITV ≥ 35 N/cm	Mann–Whitney U test	
	N = 11	N = 25	Z	*P*
Coronal BMDC	2.23 (1.98, 2.38)	3.21 (2.67, 3.94)	− 4.173	< 0.001
Middle BMDC	2.40 (2.00, 2.72)	3.30 (2.55, 3.80)	− 2.421	0.015
Apical BMDC	2.51 (2.06, 3.34)	3.07 (2.56,3.57)	− 0.944	0.345
Total BMDC	2.30 (2.06, 2.85)	3.18 (2.66, 3.64)	− 2.799	0.005

## Discussion

ISQ and ITV are important indicators for evaluating implant stability^12^. Preoperative estimation of primary implant stability is crucial to mitigate excessive micro-motion during early recovery stages^13^. However, consensus on the relationship between ISQ and BMD, ITV and BMD remains elusive. Preoperative assessment of primary implant stability is vital to prevent undue micromotion in the initial healing phase. Therefore, we proposed that the AI-based BMD grading system could offer reference for predicting ISQ and ITV, thereby augmenting clinical decision-making and the efficacy of implant procedures.

The correlation between ISQ value and BMD has been controversial. Huang et al.^14^ reported that BMD was not a factor affecting the ISQ value of primary implant stability for reasons that in addition to the density and quality of bone in the implant site, the primary implant stability was also affected by the process of implant cavity preparation, the type of implant used, and treatment of surface and geometric design of implants^15^. Harikrishnan et al.^16^ pointed out that sites with higher bone density (such as mandibular anterior teeth and mandibular posterior teeth) showed higher primary stability, and that the higher the HU value was, the higher the primary implant stability. Strub et al.^17^ also pointed out that the primary implant stability is the result of the compression of the local bone in the implant site, which is related to the mechanical interlocking between the bone and implant, and the quantity and quality of the local bone and other factors. Therefore, in the following studies, the BMD of the implant site has been proved to affect the ISQ value proportionally, that is, the higher the local BMD, the higher the ISQ value after the implant^18,19^.

In this study, the correlation between ISQ and jaw BMD was analyzed, and there was a moderately significant correlation between ISQ and the total BMDC around the implant, which was consistent with the results of other studies. In an in vivo study, Merheb et al.^20^ found that cortical bone thickness, HU value and ISQ of different sites had certain correlation with damping capacity assessment (Periotest), and proposed that implant stability could be predicted according to preoperative radiological parameters. In a study by Al-Jamal et al.^21^ there is a significant correlation between the HU value around the implant from the preoperative CBCT obtained with the use of implant design software and the ISQ recorded after implantation. Consistent with this view, Salimov et al.^22^ also noted that there was a significant correlation between BMD and ISQ among all clinical variables. It is clear that considering the BMD of the implant site before the operation is helpful in assessing and predicting the primary implant stability^23^.

Our study segmented the implant region into coronal, middle, and apical sections to analyze the ISQ-BMDC relationship in these specific areas. In both Straumann and Nobel implant group, a strong correlation was observed between ISQ and the coronal BMDC, while ISQ and the apical BMDC showed no significant correlation. Supporting our observations, Chatvaratthana et al.^24^ also observed a strong correlation between ISQ and apical cortical bone thickness in a study involving 19 implants from 16 patients. They also noted a correlation between ISQ and cortical bone thickness on the buccal or lingual side 3 mm below the alveolar crest, but not at greater depths (6–9 mm), suggesting a limited impact of apical bone on implant stability. This may be due to the limited influence of the apical bone on implant stability. It is also evident from the value of R^2^ of the regression analysis that the BMDC of the neck has the greatest effect on the ISQ values. We tried to perform a multifactorial regression equation analysis, but the model showed no significant results (*P* > 0.05). It was hypothesized that this was because the BMD values at each site were independent of each other and varied widely from site to site. It was considered that larger sample sizes and more rigorous control of experimental conditions were needed to further investigate how much of the influence of the ISQ value at a locus is contributed by coronal, middle and apical BMD, respectively. Consequently, it indicates a more pronounced correlation between coronal BMD and post-implantation ISQ, implying that higher coronal BMD enhances primary implant stability. Clinically, this suggests incorporating BMD assessments of the coronal region during implantation procedures. In cases of high BMD, adjusting the cavity preparation process, such as employing tapping or coronal shaping, may be beneficial. Conversely, for lower coronal BMD, increasing the discrepancy between implant cavity diameter and the implant itself could enhance stability.

Due to the limitations of a torque wrench in displaying detailed ITV, our study adopted a categorization method, as recommended by scholars like Noaman et al.^25^, dividing ITVs into two groups: ≥ 35 N/cm and < 35 N/cm. This approach was necessitated when a torque wrench, lacking the capability to display precise ITV, was used for implant insertion if the implant handpiece failed to place the implant at the desired depth. Consequently, one of the limitations of this study is the absence of precise ITV data.

The relationship between ITV and Bone Mineral Density (BMD) has been subject to diverse interpretations in various studies. Hakim et al.^22^ calculated the correlation between the HU value of the implant site obtained from CBCT and ITV and other indicators. The results showed that the ITV was significantly correlated with the HU value of the implant site. In an in vitro study, Comuzzi et al.^26^ used polyurethane foam blocks at two densities, 10 pounds per cubic foot (10 PCF) and 20 PCF, and ITV and ISQ values were significantly higher in 20 PCF blocks than in 10 PCF bone blocks. Ribeiro-Rotta et al.^27^ suggested that the diversity of microstructural bone configurations is related to the complexity of structural factors. A homogenous and dense mesh of trabeculae with increased intertrabecular connectivity does not always lead to high ITV values. Nevertheless, certain studies have indicated that the association between ITV and BMD at the implant site was insignificant. Herekar et al.^28^ considered primary stability to be a mechanical phenomenon that depends on the surgical technique and implant design. In our study, both Straumann and Nobel implant groups found a significant increase in BMDC in the coronal with an ITV ≥ 35 N/cm compared to those with an ITV < 35 N/cm. This finding suggested that the primary implant stability is greatly influenced by the stiffness and structural integrity of the adjacent bone during surgical procedures^12^. Improving ITV may result in significant benefits such as reducing micromotion and improved implant-bone integration. Notably, in the Nobel implant group, implants with a ITV ≥ 35 N/cm demonstrated significantly higher BMDC compared to those with a ITV < 35 N/cm. However, in the Straumann group, BMDC did not significantly differ across ITV categories, potentially attributable to the macroscopic design of Nobel implants. The midsection of Nobel implants, with its conical shape, likely exerts additional force during insertion, thereby increasing ITV.

**Figure 9:**
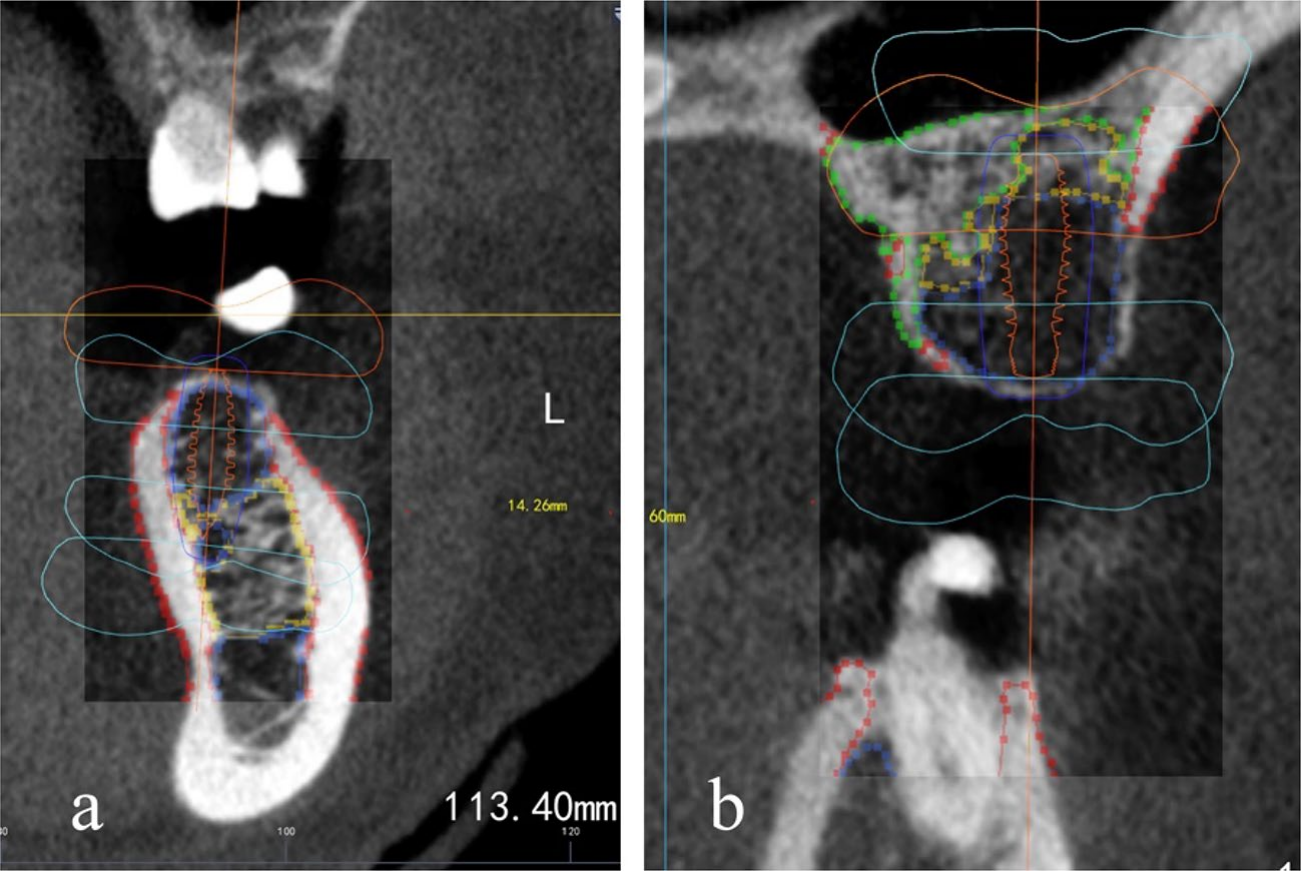
(**a**,**b**) BMD differed between buccal and lingual of the implant site.

Success of implant restoration depended on various factors such as the cortical thickness, the structure of the trabecular bone and the anatomical composition around the implant^23^. Hence, it is crucial to evaluate the BMD around implants prior to surgery^29^. Presently, clinical practice lacks a clear consensus on the definition of bone quality. Typically, this concept encompasses factors like the bone mineralization degree and bone trabecular morphology. The most extensively recognized classification of bone quality to date is the classification proposed by Lekhoolm and Zarb^6^. This classification categorized the bone into four groups based on the proportion of compact and cancellous bone. However, this classification broadly categorized bone and did not delve into the analysis of bone density variations at specific regions, failing to accurately represent the true condition of the bone. Notably, bone density varies at different sites such as the coronal and apex, mesial and distal middle of the implant, and adjacent site might show significant variations in density. The primary aim of bone classification has been to equip surgeons with a comprehensive understanding of the implant site, thereby aiding in the selection of suitable drilling tools, implant diameters, and surgical procedures to ensure optimalprimary implant stability. With the new AI-based BMD grading system, a better classification of bone density was possible (Fig. 9)^12^. This system enables the surgeon to understanded the specific density in different implant site, bridging the gap left by traditional classification methods. To facilitated statistical analysis, the BMDC generated by the new AI-based BMD grading system were utilized in this study. The BMDC correlates with varying BMD grades and the proportion of BMD within 1 mm proximity of the implant. In our study, the peri-implant BMD was segmented into three parts: the coronal, the middle and the apical regions with their BMDC calculated respectively. All of the results mentioned aboved indicate that the BMDC calculated based on the new AI-based BMD grading system can serve as a dependable indicator for assessing actual bone mass and as a predictor of primary stability. The system provides the clinician with an indication of localized BMD inconsistencies at the implant site. In clinical situations, inconsistencies in bone density can lead to drill deflection or localized thermal damage during surgery, which can subsequently affect the precision of implant placement and the stability and effectiveness of the implants. The BMDC, serving as a quantitative indicator for the validation of the new AI-based BMD grading system, effectively confirms the correlation between BMD, ISQ, and ITV. The BMDC can thus be instrumental in aiding surgeons with preoperative decision-making, enabling them to select the most suitable drill spot and and speed for precise implant positioning. Broadly defined bone tissue characteristics often lead to inconsistent classifications across studies, posing challenges in comparing and applying results to practical clinical scenarios. Therefore, caution should be exercised in interpreting and extrapolating results due to the challenges of bone type classification in the existing literature^27^. Given these complexities, it is imperative to conduct further research to establish BMDC as a direct, practical, and effectivetical, and effective.

## Conclusions

Our study revealed a positive correlation between the Bone Mineral Density Coefficient (BMDC) and primary implant stability. The BMDC of the coronal and middle bone surrounding the implant demonstrated a significant positive correlation with primary implant stability. The BMDC at the apical site of the implant did not exhibit a significant correlation with primary implant stability. The BMDC, as determined by the AI-based Bone Mineral Density (BMD) grading system, offers a more precise representation of BMD distribution at the intended implant site. This advancement provides a robust and reliable reference for predicting the primary implant stability.

## Data Availability

The datasets analyzed during the current study are available from the corresponding author on reasonable request.
